# Letter to the Editor From Spence: [Prevalence and Characteristics of Low-Renin Hypertension in a Primary Care Population]

**DOI:** 10.1210/jendso/bvae147

**Published:** 2024-08-27

**Authors:** J David Spence

**Affiliations:** Neurology & Clinical Pharmacology, Western University, and Director, Stroke Prevention & Atherosclerosis Research Centre, Robarts Research Institute, London, ON, Canada N6G 2V4

There is an important conceptual omission in the review of low-renin hypertension by Shah et al [1].

In focusing on mineralocorticoid receptor antagonists as the appropriate treatment for low-renin hypertension, they ignore the important existence of 2 streams of low-renin hypertension: low renin/high aldosterone and low renin/low aldosterone. The former represents a primary aldosteronism (PA) phenotype, the latter a Liddle syndrome phenotype, with excess activity of the renal epithelial sodium channel.

The reason this is important is that the treatment is different: mineralocorticoid antagonists are the appropriate treatment for the PA phenotype, whereas amiloride is the appropriate treatment for the Liddle phenotype [2]. (Triamterene, a possible alternative, is probably nephrotoxic [3]).

A PA phenotype is common, as variants of at least 6 genes contribute to hyperaldosteronism. (*CYP11B2*, *KCNJ5*, *ATP1A1*, *ATP2B3*, *CACNA1D*, and *ARMC5*) [4]. Although true Liddle syndrome due to variants of *SCNN1B*/*ENaC* is rare, there are polymorphisms of at least 5 other genes that result in the Liddle phenotype: *GRK*, *NEDD4L*, *CYP4A11*, *NPPA*, *UMOD*, and perhaps others [4].

A Liddle phenotype is far more common than most physicians suppose. In a study in China [5], “Among patients with systolic pressure ≥180 mmHg ± diastolic pressure ≥ 100 mmHg stimulated only by diuretics, the phenotypes were 25% Liddle, 38% Primary Aldosteronism, 8.7% renal, and 28.3% mixed.” [5]. In the Jackson Heart Study in Mississippi, “15.9% had the Liddle phenotype and 9.3% had the PA phenotype. Amiloride would therefore have been the appropriate therapy for 1.68-fold more patients than an aldosterone antagonist. This is why simply adding an aldosterone antagonist in patients with uncontrolled hypertension, as recommended in guidelines, would miss an important opportunity for better BP control” [6].

## Abbreviation

PA: primary aldosteronism
